# Clinical Significance of Hyperhomocysteinemia in Free Flap Failure: A Case Report

**DOI:** 10.1055/a-2336-0262

**Published:** 2024-08-06

**Authors:** Abeje Brhanu Menjeta, Hyung Hwa Jeong, Tae Hyung Kim, Seongsu Jeong, Changsik John Pak, Hyunsuk Peter Suh, Joon Pio Hong

**Affiliations:** 1 Division of Plastic and Reconstructive Surgery, Department of Surgery, St. Paul's Hospital Millennium Medical College, Addis Ababa, Ethiopia; 2 Department of Plastic and Reconstructive Surgery, University of Ulsan College of Medicine, Asan Medical Center, Seoul, Korea

**Keywords:** hyperhomocysteinemia, free flap failure, hypercoagulability

## Abstract

Failure of a microvascular free flap remains rare, yet multiple failures can occur, particularly in the presence of hypercoagulable conditions. This case series highlights our experience with a rare hypercoagulable state: hyperhomocysteinemia.

We present two cases of patients with hyperhomocysteinemia in this report. High-dose heparinization was administered to both patients, resulting in successful salvage of one flap and failure of the other. Notably, one patient had a history of prior free flap failures. However, after correcting hyperhomocysteinemia, subsequent free flaps were successful.

In cases of detected complications, a coagulability study is warranted, and adjustments to anticoagulation treatment may be necessary. Furthermore, when a history of flap failures is evident, screening for hyperhomocysteinemia may be warranted, with correction made prior to reconstruction.

## Introduction

Microvascular free flap failure is becoming relatively rare in high-volume centers as an anastomosis technique and the surgeon's experience accumulates with volume. 1 Nevertheless, flap complications and failures will continue to exist due to the complexity of the surgery and the multiple variables involved despite the evolved technique and experience. Failure and complication can be single or multifactorial including hypercoagulability, infection, hypotension, malnutrition, vasopressors, type of free flap used, recipient vessels, and others. 2

Hypercoagulability, often undervalued, is a proven risk for vascular thrombosis and poses a major threat to microvascular free tissue transfer and reconstruction. 3 4 5 Hypercoagulability may result from various etiologies leading to an overall incidence of thrombosis and flap loss as high as 13% and 10.3%, respectively. 5 One possible cause may be hyperhomocysteinemia which is rarely reported in literature in regards to free flap failures. Homocysteine physiological levels are commonly considered normal between 5 and 15 μmol/L, while hyperhomocysteinemia is considered mild when ranging from 15 to 30 μmol/L, intermediate for values between 30 and 100 μmol/L, and serious for values exceeding 100 μmol/L. 6 Hyperhomocysteinemia in a mild form is approximated to be found in 5 to 7% of the general population and 40% in patients with vascular disease. 7 Increasing evidence suggests that hyperhomocysteinemia is closely related to venous thrombosis, peripheral vascular diseases, coronary artery disease, myocardial infarction, and stroke. 6 7 8 It has also been reported that lowering a patient's homocysteine levels by 25% decreases stroke risk by 19%. 8

Despite the increasing evidence on hypercoagulopathy, little has been studied or reported regarding the possible effects of hyperhomocysteinemia on microvascular reconstruction. 2 In this study, the authors will present work on identifying the incidence, establishing hyperhomocysteinemia as a possible risk factor for flap complication, and providing insights on the management of such a condition.

## Case

A retrospective chart review was conducted from December 1, 2021, to December 1, 2022, revealing two cases of hyperhomocysteinemia patients. Patient consent forms were obtained for the use of photographs and information in the study.

### Case 1

**Fig. 1:**
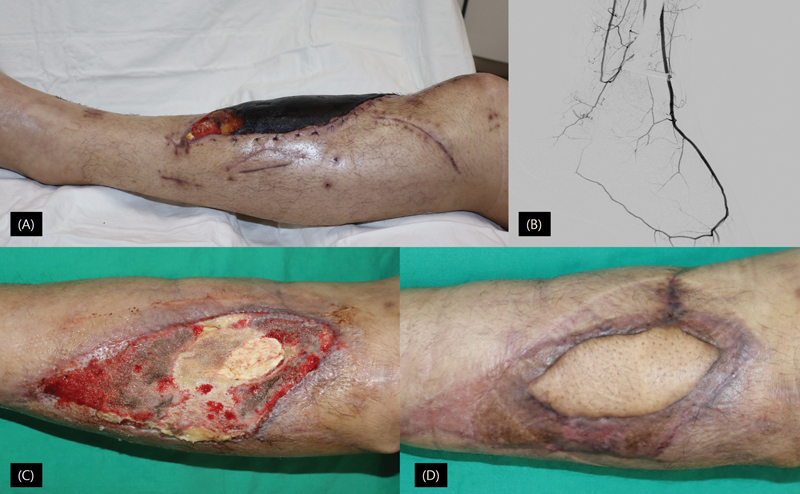
. ( **A** ) Initial photography of the case report during the initial evaluation at our center. ( **B** ) Conventional angiography of the case report. ( **C** ) Postop second-week picture of the case report after failed SCIP free flap. ( **D** ) Postop second-month picture of the case report following MSAP free flap. MSAP, medial sural artery perforator; SCIP, superficial circumflex iliac artery perforator.

A 39-year-old male patient presented with an open tibiofibular fracture in his left leg, accompanied by a soft tissue defect, 7 months postinitial injury. Despite three prior failed attempts at free flap reconstruction (two anterolateral thigh [ALT] flaps and one thoracodorsal artery perforator flap), the patient's condition worsened, manifesting as a necrotic flap covering the anterior middle third of the left leg, equinus deformity, and restricted knee joint motion ( Fig. 1 ). Notably, the patient had no comorbidities, with three major arteries intact, but exhibited elevated homocysteine levels (42.1 μmol/L) on coagulation studies ( Fig. 1 ).

Given the unsuccessful previous attempts, postoperative heparin infusion was planned following consultation with hematology. Reconstruction was attempted using a superficial circumflex iliac artery perforator (SCIP) free flap connected end-to-side on the anterior tibial artery (ATA) and end-to-end on the accompanying vein. Unfortunately, signs of flap distress emerged on the first day, necessitating exploration. Thrombosis was discovered in both the artery and vein, prompting thrombectomy, irrigation with heparin, and reanastomosis. Despite these interventions, flap perfusion weakened within 30 minutes, and further thrombosis occurred, prompting a change in the recipient pedicle to the posterior tibial artery (PTA) and vein using vein grafts. However, thrombosis recurred in the vein graft despite intensive heparinization. Consequently, the free flap was abandoned, and a skin graft harvested from the SCIP flap was applied over the defect ( Fig. 1C ), with negative pressure wound therapy employed.

Subsequent intervention focused on normalizing the elevated homocysteine level through folate and vitamin B12 supplementation, resulting in a reduction to 14.1 μmol/L over 4 months. A second attempt at free flap reconstruction using the Medial Sural Artery Perforator technique on the ATA and accompanying vein proved successful, without complications ( Fig. 1D ).

### Case 2

**Fig. 2.**
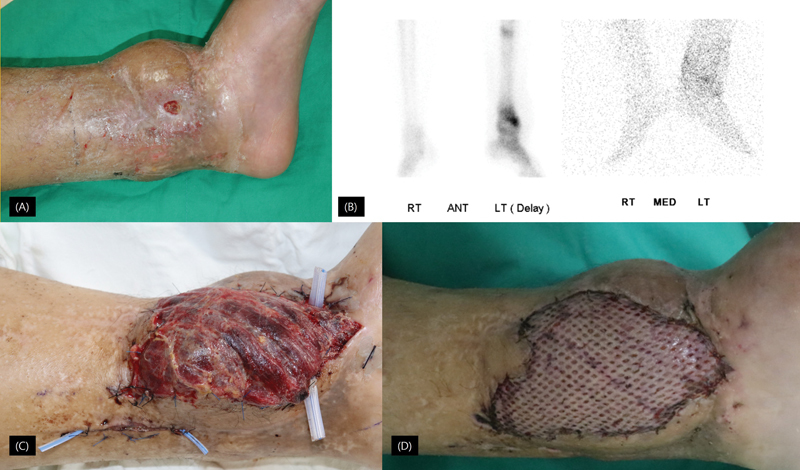
( **A** ) Initial photograph of the patient presenting a skin defect with exposed bone due to chronic osteomyelitis. ( **B** ) Bone (left) and WBC (right) SPECT/CT imaging of the patient demonstrates a nonunion in the left distal tibia, characterized by diffusely increased tracer uptake, sclerotic changes, and WBC accumulation around a comminuted fracture with a dislodged screw, suggestive of probable osteomyelitis. ( **C** ) Photograph taken on the fifth postoperative day after gracilis muscle flap reconstruction in the patient with chronic osteomyelitis and a history of free flap failure. ( **D** ) The application of a skin graft following stabilization of the free flap.

A 29-year-old male patient presented to our hospital 17 months after sustaining a road traffic accident, resulting in an open distal tibiofibular fracture and a soft tissue defect on his left ankle. He had no history of diabetes or hypertension but had previously undergone a failed ALT free flap reconstruction, performed 1 month after the accident. On examination, an open wound was observed on the medial side of the left ankle, with exposed bone and serous discharge ( Fig. 2 ). Further investigation revealed a comminuted fracture with chronic osteomyelitis and nonunion of the left distal tibiofibular region, confirmed by bone spectroscopy. Additionally, his coagulation profile showed an elevated serum homocysteine level of 26.8 μmol/L.

To address the chronic osteomyelitis and soft tissue complications, a gracilis muscle flap (13 cm × 6 cm) based on the medial circumflex femoral artery and accompanying vein was performed. The PTA and accompanying vein were utilized as recipient vessels, with an interposition vein graft harvested from the great saphenous vein. Despite successful microvascular anastomosis of one vein and one artery to the PTA, flap congestion was noted on the same day, necessitating a return to the operating room. A nonfunctional venous anastomosis was identified, leading to irrigation with heparin followed by microanastomosis revision using another vein graft.

Unfortunately, nonreassuring flap monitoring outcomes on the first postoperative day prompted further revision, revealing a clot in the artery and kinking of the vein graft. The vein grafts were excised, and one vein and one artery were reanastomosed, with aggressive heparinization initiated. The wound at the revision site was closed primarily, and the ankle was splinted at 90 degrees. Heparinization was continued for 2 weeks.

By the third postoperative week, heparinization was tapered based on coagulation profile results. The gracilis muscle flap was then covered with split-thickness skin grafts. In the fifth postoperative week, the patient attended an outpatient appointment, where a well-taken muscle flap and split-thickness skin graft on the medial aspect of the left ankle were observed. Partial weight-bearing was permitted based on these findings, and the patient was scheduled for subsequent follow-up appointments.

## Discussion

Hyperhomocysteinemia has been implicated in various health conditions, including hypercoagulability, thromboembolism, peripheral vascular diseases, coronary artery disease, myocardial infarction, and stroke, as evidenced by epidemiological studies. 2

Homocysteine, a sulfhydryl-containing amino acid, is derived from the metabolism of methionine, an essential amino acid. In healthy individuals, excess homocysteine in the bloodstream is typically metabolized via either a remethylation pathway to methionine or a transsulfuration pathway to cysteine. 9 However, when these metabolic pathways are impaired, excess homocysteine can undergo auto-oxidation, leading to the formation of biologically reactive products that induce cellular toxicity and contribute to blood clot formation in arteries and veins. 9 The plasma level of homocysteine is influenced by both genetic and environmental factors. Genetic defects in genes encoding enzymes such as Methyltetrahydrofolate reductase and Methionine synthase can cause hyperhomocysteinemia. Additionally, environmental factors such as dietary deficiencies of vitamin B12, B6, and folate, as well as impaired renal function, can elevate plasma homocysteine levels. 9 10 11 Consequently, treatment often involves supplementation with vitamin B and folic acid. In cases where multiple flap attempts failed despite immediate postanastomosis high-dose heparin treatment, a similar protocol using vitamin B and folic acid supplementation for 4 months successfully normalized homocysteine levels. This normalization ultimately led to a successful outcome after microsurgery.

Hyperhomocysteinemia is diagnosed when plasma homocysteine levels exceed 15 μmol/L, with phenotypes varying depending on severity: mild (15–24 μmol/L), moderate (25–100 μmol/L), or severe (>100 μmol/L). The average normal plasma homocysteine level is approximately 10 μmol/L. 12 Mild hyperhomocysteinemia is estimated to affect 5 to 7% of the general population globally and up to 40% of patients with vascular disease. Smoking and betel nut use have been associated with a higher prevalence of hyperhomocysteinemia. 13 14 15 16 17 18

The incidence of mild hyperhomocysteinemia in this series was approximately 2% (3 out of 126 cases), lower than the general population rate of 5 to 7%. 7 Despite this, even a 2% incidence rate still poses a potential risk for complications after free flap surgeries. The authors initially overlooked the possibility of hyperhomocysteinemia as a high-risk factor for thrombosis and did not consider preoperative correction. In this report, we present three cases of patients with elevated homocysteine levels ( Table 1 ).

**Table 1:** List of cases with an early free flap complication in patients with hyperhomocysteinemia

Number	Age (years)	Sex	Free flap type	Type of free flap complication	Homocysteine level at the time of failure	Total attempt of failures
1	39	M	SCIP	Venous thrombosis	42.1 μmol/L ↑	4
2	30	M	Gracillis	AV thrombosis	26.8 μmol/L ↑	1
3	29	M	SCIP	Arterial occlusion	25.8 μmol/L ↑	0

Abbreviation: SCIP, superficial circumflex iliac artery perforator.

Coagulation index studies postcomplication often appear normal and do not indicate any hypercoagulative factor. However, our study suggests a significant subset of patients with hyperhomocysteinemia, strongly associated with postmicrosurgery complications, warranting the inclusion of homocysteine level evaluation in coagulation index studies. Additionally, when dealing with patients who have experienced previous flap failures or thrombotic episodes, obtaining preoperative homocysteine level studies may elucidate reasons for failure and allow for corrective measures to prevent further complications. Of the three patients with hyperhomocysteinemia in our series, two had experienced prior failures.

For patients diagnosed with hyperhomocysteinemia, anticoagulant medications such as aspirin, clopidogrel, heparin, and warfarin are recommended to prevent blood clots and treat microvascular thrombosis. 2 9 In two of the three patients with hyperhomocysteinemia, high-dose heparin was successful in providing adequate circulation, leading to successful flap outcomes. Our protocol includes a 5,000 IU bolus of heparin followed by continuous infusion of heparinized saline solution (500 mL of normal saline with 20,000 IU of heparin) at a rate of 2 mL/h, targeting an activated partial thromboplastin time (aPTT) level of 60 to 80 for 2 weeks postsurgery. Adjustments to the infusion rate are made based on aPTT levels. However, one patient with an intermediate level of 42.1 μmol/L did not respond to heparin and experienced failure. Further research is needed to determine the optimal heparin dosage based on the severity of hyperhomocysteinemia-induced thrombosis and to develop an algorithm for flap complications related to hyperhomocysteinemia.

Several treatment options are globally recognized and readily available for hyperhomocysteinemia, including folic acid, vitamin B12, and pyridoxine. Studies have shown that folic acid or pyridoxine alone can reduce plasma homocysteine levels by 22% while administering both together can lead to a 38.5% reduction. Vitamin B12 alone can result in an 11% reduction in plasma homocysteine levels. 19 Randomized clinical trials have demonstrated a significant decrease in plasma homocysteine levels with oral supplementation of a combination of folic acid, vitamin B6, and vitamin B12. 20 Research has also shown that a 1-month treatment of hyperhomocysteinemia with pyridoxine (600 mg/d) and folic acid (10 mg/d) can partially reduce homocysteine levels. 21 Small doses of folate (0.4–5 mg/d), vitamin B12 (400 μg/d), and vitamin B6 (10–50 mg/d) have been suggested to rapidly decrease plasma homocysteine concentration. Furthermore, a combination supplementation of folic acid (25 mg), vitamin B6 (25 mg), and vitamin B12 (250 μg/d) has been associated with a reduction in atherosclerosis, as evidenced by decreased carotid plaque.

It is important to note that the timing of these treatments to meals is crucial. Pyridoxine is most effective when taken after a meal, while folic acid and vitamin B12 primarily act under fasting conditions. Adjusting the timing of intake accordingly is necessary for optimal efficacy. 22

### Conclusion

When complications arise during free flap monitoring, it is essential to conduct a coagulability study and adjust anticoagulation treatment accordingly. Additionally, in cases where a history of flap failures is evident, screening for hyperhomocysteinemia may be necessary, with corrections made before proceeding with reconstruction.
